# Data on ultrasound therapy as an adjuvant pain control method among Indian TMDS patients

**DOI:** 10.6026/97320630018774

**Published:** 2022-09-30

**Authors:** Nidhi Mishra, Prajakta Barapatre, Madhu Pandey, Hiroj Bagde, Gagandeep S Randhawa, Abhishek Balani, Kapil Paiwal, Ramanpal S Makkad

**Affiliations:** 1 Department of Oral Medicine and Radiology, Bhabha Dental College, Bhopal (M.P), India; 2 Department of Prosthodontics and Crown and Bridge, Mahatma Gandhi Dental College and Hospital, Jaipur, (Rajasthan), India; 3 Department of Orthodontics and Dentofacial Orthopedics, MCDSRC, Durg, India; 4 Department of Periodontology, Rama Dental College and Research Centre, Kanpur, UP, India; 5 Department of Oral and Maxillofacial Surgery, Ahmedabad Dental College and Hospital, Ahmedabad, India; 6 Department of Oral and Maxillofacial Surgery, New Horizon Dental College & Research Institute, Sakri, Bilaspur Chhattisgarh, India; 7 Department of Oral Pathology and Microbiology Daswani Dental College & Research Center, Kota, India; 8 Department of Oral Medicine and Radiology, New Horizon Dental College and Research Institute, Sakri, Bilaspur Chhattisgarh, India

**Keywords:** Ultrasound, temporomandibular disorders

## Abstract

It is of interest to evaluate the efficacy of ultrasound therapy as an adjuvant pain control modality in dysfunctions of the temporomandibular joint. The study comprised 20 patients with TMJ issues who had received a clinical diagnosis of temporomandibular disorders (TMDS). These patients underwent independent VAS evaluations for the intensity of pain, opening and closing of the mouth, and soreness of the muscles of mastication, including the masseter muscle, medial pterygoid muscle, lateral pterygoid muscle, and temporalis muscle, as well as additional auxiliary muscles. The chosen patients received ultrasonic treatment. The mean value of mouth opening before therapy was 39.51cm, with SD values of 7.61 cm. The mean value of mouth opening after therapy was 42.91 cm with SD values of 6.08.The findings were statistically significant, with a p-valueof0.021. The mean value of VAS in the TMJ area before therapy was 8.41 with SD values of 2.11.There was a reduction in the mean values of VAS after therapy, which was 3.11 with SD values of 1.12. The findings were significant statistically, with a p-value equal to 0.001. Thus, ultrasonographic therapy for temporomandibular joint pain demonstrated a considerable improvement in pain reduction and mouth opening. It is possible to view this therapy as the adjuvant methodology to control pain in disorders of TMJ.

## Background:

The term "temporomandibular disorders" (TMDs) refers to a group of general health-related and dental-related disorders that impact the muscles contributing to the mastication of food and associated soft tissue elements. They are therefore considered a musculoskeletal disease of masticatory assembly of the head and neck [1]. Besides, TMDs may also accompany additional neurological symptoms such as headaches, vertigo, heaviness, and vision abnormalities [2]. The primary treatment goals for all TMD patients are pain relief, the return of normal jaw function, and maintaining a normal way of life [3]. Numerous therapeutic modalities can be used to manage these disorders, including conservative therapy, behavioural therapy, physiotherapy, pharmaceutical therapy, and occlusal appliances [4]. Sonography (US), microwave therapy, low-level laser application of Lasers, and TENS are examples of electro physical therapies [5]. The same emitting gadget reads the returned sound waves and converts them into images [6]. Different components in the TMJ complex return sound waves in different ways. While the edge of the bone is hyperechoic because of the high rebound of sound waves) and looks white in ultrasound pictures, tissue of bone, found in the condylar head of the mandible and temporal bone articular eminence, is often hypoechoic in nature because of reduced reflection of sound waves) [7]. A hyper echoic (white) streak is produced because of the joint's capsule and the muscles' surface as they both strongly reflect the sound waves [8]. Due to the contact between the opposing surfaces, these anatomic spaces are imaginary and typically undetectable until there is an effusion [9-10]. In evaluating the closed mouth position findings, the disk's position is considered normal if the disk's intermediate zone is located between the antero superior aspect of the mandibular condyle and the postero inferior aspect of the articular eminence [11]. Ultrasound therapy physical therapy is one such efficient method for managing disorders of temporomandibular joints, which are the latest emerging methods for their treatment [12]. Therefore, it is of interest to assess the benefit of therapy through ultrasound in patients suffering from temporomandibular joint disorders.

## Methods and Materials:

A maximum of twenty patients between 18 and 50, of either gender, were enrolled in the study. TMJ issues were clinically assessed in patients utilizing research diagnostic standards (RDC). The study comprised 20 patients with TMJ issues who had received a clinical diagnosis. Patients having benign lesions and malignant lesions of disorder of temporomandibular joint, individuals with unexplained toothache, individuals with lesions over the skin or facial scratches at the spot of placement of the acoustic gel, pregnant women, individuals having cardiac pacemakers and suffering from cardiac arrhythmias, individuals having a history of trauma of temporomandibular region, individuals suffering from ankylosis of TMJ were not included in this research Using a visual analogue scale, the pain was assessed (VAS). With a written consent form, data were entered into the case proforma. These individuals underwent a radiographic evaluation to rule out odontogenic infection and bone abnormalities in the condylar region. These patients underwent independent VAS evaluations for the intensity of pain, opening and closing of the mouth, and soreness of the muscles of mastication, including the masseter muscle, medial pterygoid muscle, lateral pterygoid muscle, and temporalis muscle, as well as additional auxiliary muscles. The chosen patients received ultrasonic treatment. For four comparable weeks, this therapy was given once a week. The frequency used was one MHz. The pulse setting was adjusted at 1:1. Duration of therapy was 8 minutes per session. Upon the fifth visit following treatment, VAS was used to assess each patient's pain pattern and the soreness of their muscles involved in masticating food. Data were afterward tabulated, and statistical analysis was performed on them.

## Statistical analysis:

Data were analyzed using the SPSS and reported as mean standard deviation. Paired sample t-tests were used for pain-free IID comparisons within groups. For comparisons of inter group pain before therapy and four weeks after treatment, independent t-tests were conducted. p≤0.05 was regarded as statistically significant.

## Results:

In this study, 9 participants were males, while 11 were females. 45% of the study participants were males, while 55% were females. The mean age of study participants was 33.71 ±13.71 years, while the study participants were in the range of 21 to 50 years (Table 1 - see PDF). There was an evaluation of opening of mouth before therapy and after therapy in all study participants. The mean value of mouth opening before therapy was 39.51cm, with SD values of 7.61 cm. The mean value of mouth opening after therapy was 42.91cm, with SD values of 6.08.The findings were statistically significant, with a p-value of 0.021 (Table 2 - see PDF). The mean value of VAS in TMJ before therapy was 8.41, with SD values of 2.11.There was a reduction in the mean values of VAS after therapy, which was 3.11 with SD values of 1.12. The findings were significant statistically, with a p-value equal to 0.001 (Table 3 - see PDF). There was the evaluation of mean values of VAS score in ultrasonic therapy in the masseter region. The mean value before therapy was 9.11, with SD values of 1.24. There was a reduction in the mean values after therapy, which was found to be 4.11 with an SD of 1.34. The decrease in the mean VAS values during ultrasonic therapy in the masseter region was statistically significant (Table 4 - see PDF). The mean VAS score before therapy was 8.65 with a standard deviation value of 2.31, while the mean VAS score after therapy was 2.67 with a standard deviation value of 1.41. There was a significant reduction in the mean values of VAS, showing a reduction in tenderness in the temporalis region (p≤ 0.05) as shown in Table 5(see PDF).

## Discussion:

The individual's needs should precede the disorder while deciding on a TMD treatment plan. Although different therapy modalities comparably alleviate pain and functionality, caution is advised when using traumatic and other irrevocable treatments, especially when treating TMD patients for the first time [14]. Physical therapy aids in musculoskeletal pain relief and functional recovery. It can lessen an innate discomfort, lead to a lot bigger opiate release, and aid in more profound pain suppression [13]. Physical therapy has the potential to reduce musculoskeletal discomfort and return the body to its normal state. It lessens discomfort naturally, improves opiate release, and aids in achieving more severe pain control without any negative side effects. Both thermal effects as well as and non-thermal effects are a part of this treatment approach [15]. It may cause a rise in regional blood flow, decrease muscular spasms, and improve collagen fiber extensibility. De-lamination and sonic micro-streaming are examples of non-thermal processes that promote the repair of fibroblasts and the synthesis of collagen, tissue regeneration, and bone healing. Significant improvement was seen throughout this ultrasound-guided procedure. Therefore, it can be utilized to manage TMD. It can be a non-invasive and cost-effective therapy [16].

Several studies have indicated that ultrasound is useful in treating TMDs, in accordance with the literature that is currently available. With a mean age of 33 years, this study displayed the age distribution from the second to the fourth decades of life. According to a survey conducted by Gray RJ et al., the age distribution was similar to our study's [6]. Data showed a female predominance comparable to the Geissler and McPhee study [7]. TMD is most frequently caused by psychological stress or extended work in patients. After comparing the maximal mouth opening measurements taken before and after treatment, it was discovered that there is a statistically significant elevation in the maximum opening of the mouth. In between therapy, the decrease in the values of VAS for pain at the TMJ region was shown to be highly statistically relevant (P≤0.05). Speed et al. noted that the ultrasound group demonstrated a greater success rate in pain alleviation and concluded that ultra sound acts as a pain relief mechanism for individuals with TMDs [8]. According to research by Grieder A et al., ultrasound is more helpful at treating muscular symptoms while being less beneficial at treating disc-related symptoms [9]. It has been shown by several researchers (Ucar et al.) that certain activities, including active stretching as well as passive stretching, calming exercise, isotonic tension, and health education, are useful and helpful in enhancing mandibular motions and enhancing mouth opening [10]. Like this, Laat et al. observed that these conservative and mechanical therapies significantly improved jaw function and pain metrics in individuals experiencing myofunctional pain [11]. In a related study by Fouda, it was discovered that patients undergoing US therapy opened their mouths. US therapy is, therefore, a form of complementary therapy [12]. It has long been recognized that ultrasound has the power to affect tissue and cause biological changes. Due to the absorption of ultrasonic waves, which raises tissue temperature and increases blood flow, the effects are partially thermal.

There is a mechanical impact since sound waves cause alteration in pressure in the soft tissues, creating a "micro" that is believed to improve collagen tissue elasticity and disintegration of fibrous tissue. Additionally, there is an elevation in the permeability of cells and tissues. In TMD, ultrasound is most effective at easing symptoms related to the muscles and least effective at easing symptoms related to the disc. Esposito et al. (1984) assessed the efficacy of ultrasonography in managing 28 patients. They concluded that therapeutic ultrasonography could successfully relieve MPDS discomfort when occlusal splint therapy is ineffective [17]. In this study, 9 participants were males while 11 were females. 45% of the study participants were males, while 55% were females. The mean age of study participants was 33.71±13.71 years, while the study participants were 21 to 50 years old. There was an evaluation of opening of mouth before therapy and after therapy in all study participants. The mean value of mouth opening before therapy was 39.51cm, with SD values of 7.61 cm. The mean value of mouth opening after therapy was 42.91 cm, with SD values of 6.08.The findings were statistically significant, with a p-value of 0.021. The mean value of VAS in TMJ before therapy was 8.41, with SD values of 2.11.There was a reduction in the mean values of VAS after therapy, which was 3.11 with SD values of 1.12.

The findings were significant statistically, with a p-value equal to 0.001. There was the evaluation of mean values of VAS score in ultrasonic therapy in the masseter region. The mean value before therapy was 9.11, with SD values of 1.24. There was a reduction in the mean values after therapy, which was found to be 4.11 with an SD of 1.34. The decrease in the mean VAS values during ultrasonic therapy in the masseter region was statistically significant. The mean VAS score before therapy was 8.65 with a standard deviation value of 2.31, while the mean VAS score after therapy was 2.67 with a standard deviation value of 1.41. There was a significant reduction in the mean values of VAS, showing a reduction in tenderness in the temporalis region. (p ≤ 0.05). Temporomandibular disorders are a class of general health- and dental-related conditions that affect the muscles involved in food mastication and related soft tissue components. They are consequently regarded as musculoskeletal disorders of the head and neck's masticatory assembly [18]. TMDs are characterized by a variety of symptoms, including discomfort in the orofacial region, muscle tenderness, restricted jaw motion, noise at the joint, inhibited jaw function, alteration or diversion, rigidity, pain or lethargy in the muscles of the face, and locking brought on by muscle spasm. Additional neurological symptoms such as headaches, vertigo, heaviness, and altered vision may coexist with TMDs [19].

Many factors can predispose to, increase, or aggravate TMD, including muscular impulsivity, trauma, mental anguish, and malocclusion. Pain relief, the restoration of normal jaw function, and the maintenance of a normal way of life are the key objectives of treatment for all TMD patients [20].Various therapeutic techniques can be used to treat these diseases, such as conservative therapy, behavioural therapy, physiotherapy, pharmacological therapy, and occlusal appliances. TMDs are considered amenable to various physical therapies, including electro physical therapies, exercise, and physiotherapeutic methods. Physiotherapy is an essential treatment for reducing inflammation, treating myofascial pain, and regaining oral cavity motor function. Electro physical treatments include TENS, US, microwave therapy, and low-level laser application [21-24].

The impact of therapeutic sonography and TENS in treating myofascial discomfort in TMD patients was compared in a study by Rai et al. [13]. The 90 patients in this randomized comparative trial were divided into three groups, each with 30 patients: group I comprised normal control subjects, group II received therapeutic ultrasonography therapy, and group III had TENS therapy. All 90 patients had additional testing to determine the maximum inter-incisor qualitative assessment for muscle discomfort, interference with daily activities, impression of the therapy on the visual analogue scale (VAS), and frequency and intensity of the Ultrasonic therapy. Before therapy, the thickness of the masseter muscle in the patient suffering from temporo mandibular joint disorder was 13.00±1.1 mm, whereas it was 12.00±1.1 mm in the control group. In the US, statistically significant results were found for the VAS score of muscular discomfort, impairment to everyday life, and therapy impression. Following treatment, the TENS and US groups' anechoic regions decreased or vanished by 74.4 and 95.6%, respectively. It was determined that the ThUS showed up to be unquestionably better, which was associated with the VAS score of the impression of the therapy, the severity of the muscle pain, and the interference with daily activities following treatment, as well as the presence of anechoic areas.

Millions of people worldwide suffer from TMD or temporomandibular joint dysfunction. It is unknown if TMD can be effectively treated with low-intensity ultrasound (US). In a study by Ba et al. 160 TMD patients were included [14]. Two groups of participants were randomly assigned to undergo either ultrasonography therapy or no therapy. For two straight weeks, participants in the US therapy group received therapy through ultrasound once a day for five days a week. The patients' pain was evaluated using the visual analogue scale and the maximal pain-free inter-incisal separation before therapy, four weeks, and six months after the completion of therapy. In addition, assessments of cranio mandibular index considered as CMI, disability index considered as DI, and mandibular mobility (MM) were made. VAS values, IID values, MM values, JN values, DI values, and CMI values in the US group drastically improved four weeks and six months after therapy compared to values before the therapy. US group, meanwhile, had a risk of recurrence of 2.63 percent six months following therapy. US therapy is advised for TMD patients since it can dramatically relieve discomfort, enhance the functionality of the temporomandibular joint, and increase the mouth opening range. Emshoff conducted a study to evaluate the performance of ultrasound-mediated therapy over masseter muscle in TMD patients. Ten patients between 18 and 50 who met the diagnostic criteria for TMDs were included in this study. All patients had 8 minutes of ultrasound therapy weekly for four weeks, and at each visit, the pain level was assessed using a visual analogue scale. Individuals receiving therapy through ultrasonography were found to have reduced pain. TMJ pain decreased from 7.30 ± 1.70 to 4.00 ± 2.53, and the statistically significant improvement in the mouth was opening from 40.40 ± 6.50 to 41.80 ± 5.97. This treatment seems to be an effective physiotherapy technique for treating TMD discomfort.US therapy treatment can be viewed as an effective physiotherapy technique because it seems to help with pain relief and subsequent mouth opening. As a result, US therapy treatment is an effective and stand-alone therapeutic technique for TMDs [16].

To examine the effectiveness of conventional treatments against the use of both therapeutic ultrasound and conventional therapy in the treatment of patients with myofascial pain, Pereira LJ et al. conducted a study. Patients who visited complained of myofascial pain participated in the randomized comparison study. It was observed that US therapy treatment might be viewed as a helpful tool in managing myofascial discomfort because it appears to be effective in reducing pain and enhancing future mouth opening. In myofascial pain dysfunction syndrome, US therapy treatment is a powerful and independent therapeutic approach. Data is in line with that study [15]. The US has recently gained popularity as physical therapy for many illnesses. The breakdown of fibro cartilage and inordinate cell death of chondrocytes found in the soft tissue component of bone as well as temporomandibular joint as a consequence of the elevated concentration of nitric oxide and the discrepancy of metabolism in the localized region of the joint were both shown to happen as during the appearance and progression of TMD, according to earlier studies [22, 25]. It has been demonstrated that the US can lower chondrocyte apoptosis and high cytokine levels in the articular fluid. It limits the release of inflammatory cytokines and encourages fibrocartilage growth to repair the cartilage damage. In animal models, low-intensity pulsed ultrasound (LIPUS) was able to enhance mandibular growth and modify the growth of the mandible, providing additional support for US-induced mandible modification that might contribute to the observed therapeutic effect [23]. Bigger sample size would be needed for future research, and some repercussions, such as the placebo influence of this therapy, would need to be examined. This would allow for a more thorough and varied evaluation and interpretation.

## Conclusion:

Data shows that ultra sonographic therapy for temporomandibular joint pain problems demonstrated a considerable improvement in pain reduction and mouth opening. It is possible to view this therapy as an adjuvant methodology to control pain in disorders of TMJ.

## Author's contributions:

Nidhi Mishra participated in conception, design, data acquisition, and interpretation, drafted and revised the manuscript. Prajakta Barapatre participated in conception, design, data acquisition and interpretation, performed all statistical analyses, drafted and critically revised the manuscript. Madhu Pandey participated in conception, design, and critically revised the manuscript. Hiroj Bagde participated in conception, design, and critically revised the manuscript. Gagandeep S. Randhawa participated in conception, design, and critically revised the manuscript. Abhishek Balani participated in conception, design, and critically revised the manuscript. Kapil Paiwal participated in conception, design, and critically revised the manuscript. Ramanpal S Makkad participated in conception, design, and critically revised the manuscript. All authors gave their final approval and agree to be accountable for all aspects of the work.
